# Prone Position Ventilation Used during a Transfer as a Bridge to Ecmo Therapy in Hantavirus-Induced Severe Cardiopulmonary Syndrome

**DOI:** 10.1155/2013/415851

**Published:** 2013-07-15

**Authors:** R. Cornejo, D. Ugalde, O. Llanos, P. Bisbal, L. De la Barrera, C. Romero, R. Neira, Roberto González, J. Gajardo

**Affiliations:** ^1^Unidad de Pacientes Críticos, Departamento de Medicina, Hospital Clínico, Universidad de Chile, Chile; ^2^Unidad de Pacientes Críticos, Complejo Asistencial Hospital Barros Luco Trudeau, Chile

## Abstract

*Background*. Transport of critically ill patients is a complex issue. We present a case using prone positioning as a bridge to extracorporeal membrane oxygenation (ECMO), performed by a critical retrieval team from a university hospital. *Case Report*. A 28-year-old male developed fever, progressive respiratory failure, and shock. He was admitted to ICU from a public hospital, and mechanical ventilation was begun, but clinical response was not adequate. ECMO was deemed necessary due to severe respiratory failure and severe shock. A critical retrieval team of our center was assembled to attempt transfer. Prone positioning was employed to stabilize and transfer the patient, after risk-benefit assessment. Once in our hospital, ECMO was useful to resolve shock and pulmonary edema secondary to Hantavirus cardiopulmonary syndrome. Finally, he was discharged with normal functioning. *Conclusion*. This case exemplifies the relevance of a retrieval team and bridge therapy. Prone positioning improves oxygenation and is safe to perform as transport if performed by a trained team as in this case. Preparation and organization is necessary to improve outcomes, using teams and organized networks. Catastrophic respiratory failure and shock should not be contraindications to transferring patients, but it must be done with an experienced team.

## 1. Introduction

Transport of critically ill patients remains a complex issue; respiratory or hemodynamic instability increases the risks during the procedures required to transfer mechanically ventilated patients between units and particularly between ICUs at different hospitals. Interhospital transportation of critically ill patients is of paramount importance when patients require therapies not available at the hospital of origin, often life-saving measures such as extracorporeal membrane oxygenation (ECMO). In this context patients may require bridging therapy to overcome life-threatening cardiopulmonary failure for a time long enough to reach destination, performed by a specialized team, experienced in those measures.

The aim of this report is to communicate how the assessment and management by a critical care rescue team allowed the transfer of a patient with severe respiratory failure and concomitant circulatory failure in prone positioning and high dose of vasoactive drugs.

## 2. Case Report

A 28-year-old male, previously healthy, was admitted to a public hospital in Santiago because of respiratory failure and hypotension. He referred a history of 7 days of fever, progressive shortness of breath, abdominal pain, and vomiting. The patient had been camping in the foothills of the Araucanía region of Chile five weeks before the onset of symptoms. At the emergency room the patient was acutely ill with fever of 38.9°C, tachycardia of 146 beats per minute, signs of peripheral hypoperfusion, MAP of 60 mmHg, cyanosis, and tachypnea up to 40 per minute, requiring 100% oxygen to achieve and O_2_ saturation of 88%. He was transferred to the ICU and connected to mechanical ventilation. He developed rapid and progressive respiratory failure achieving PaO_2_/FiO_2_ ratio 52 mmHg, oxygenation index (OI) 34,6 (employing tidal volume of 6 mL/kg, FiO_2_ of 100%, and PEEP 24 cm of water), and hemodynamic instability characterized by high requirements of vasoactive drugs, oligoanuria, and lactacidemia over 5 mmol/L. A pulmonary artery catheter showed a cardiac index of 2.0 L/min/m^2^ while receiving norepinephrine at 2 mcg/kg/min and epinephrine 0,3 mcg/kg/min, and the SvcO_2_ was 55%. Among hematological and biochemical parameters an hematocrit of 50%, thrombocytopenia of 33.000× mm^3^, and C-reactive protein of 130 mg/L were found. X-chest ray showed bilateral and diffuse pulmonary infiltrates. After 7 hours of treatment with no evident improvement in general conditions and with the provisory diagnostic of Hantavirus cardiopulmonary syndrome, ECMO was considered the most adequate therapeutic option. As this approach was not available at the ICU, the transfer to the University Hospital ICU was evaluated.

**Figure 1:**
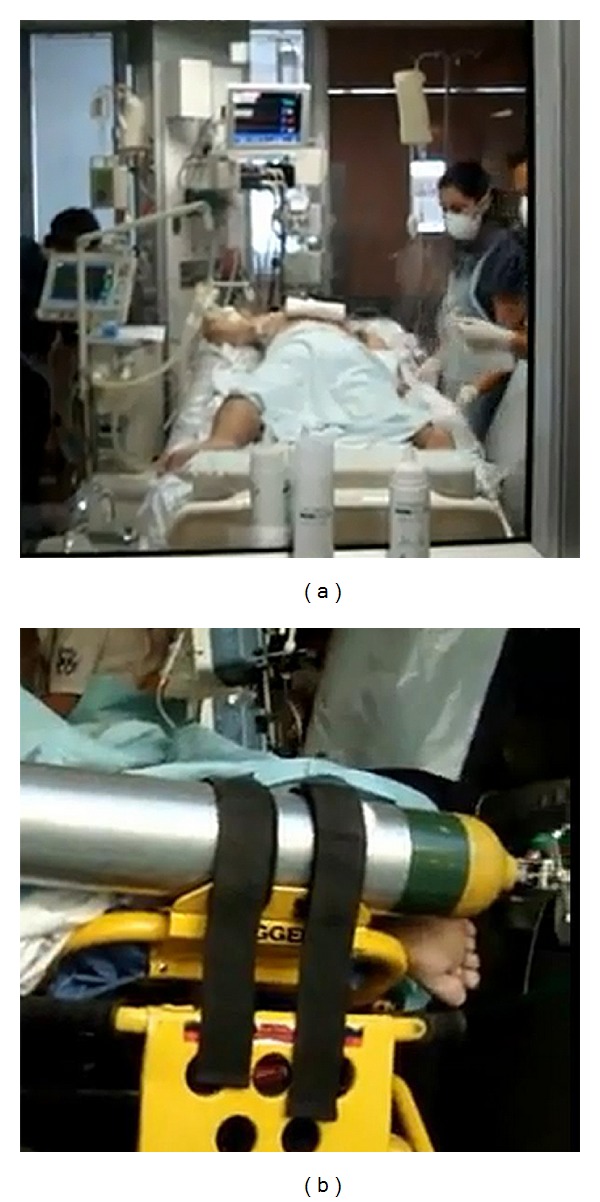
Prone position transport. Patient after prone positioning and stabilization by the retrieval team on the referring ICU (a) and during transport procedure on the ambulance where the foot orientation evidences the prone position (b).

Given the patient unstable condition with O2 saturation of 84% with 100% FiO2, pCO2 67 mmHg, and MAP 60 mmHg receiving the above-mentioned dose of vasoactive drugs, an specialized retrieval team was assembled to assess the patient and carry out the transfer. When this critical care rescue team arrived to the referring ICU the patient was stabilized and prepared for transfer. After draining abundant respiratory secretions, PEEP was lowered and vasopressors adjusted to improve CI, increasing epinephrine, decreasing norepinephrine, and setting the patient in prone position to improve respiratory function. Mechanical ventilation settings were tidal volume of 6 mL/kg, PEEP 16 cm of water, respiratory rate of 32 per minute, and 100% oxygen; the hemodynamic support based on vasoactive drugs was norepinephrine at 0.9 mcg/kg/min and adrenaline 0.7 mcg/kg/min. The team opted for transferring the patient in prone positioning as a bridge therapy to ECMO, due to the severity of shock, ARDS and what appeared to be massive capillary leakage. This decision was made in spite of the severity of the situation, and taking into account that hemodynamic as respiratory functions were partially stabilized. Additionally, the possibility of transferring the patient in ECMO was considered unviable. An ambulance was called in, and the patient was transported in prone position under the management of the critical care rescue team with no incidents or new interventions (Figure 1).

At the arrival at the University Hospital, the O_2_ saturation was 88% with 100% FiO_2_ and MAP was 72 mmHg without changes in the doses of vasoactive drugs. The patient entered immediately to the operating room for ECMO installation. During the procedure he was unstable hemodynamically and presented a large bleeding and received several transfusions (red blood cells 5 units, fresh frozen plasma 3 units, and platelets 10 units), but the procedure was completed satisfactorily.

The patient was transferred to the ICU, while on ECMO, and high volume hemofiltration was started. Among laboratory parameters a decreased fibrinogen (87 mg/dL), increased serum creatinine of 2.6 mg/dL, arterial lactate of 17 mmol/L, and thrombocytopenia of 24.000 were found. Hantavirus infection was confirmed by enzyme-linked immunosorbent assay (ELISA). Echocardiography at day 2 on ECMO revealed severe left ventricle systolic dysfunction with an ejection fraction of 26%. However, continuous ultrafiltration therapy was well tolerated, and a day after, vasopressors were suspended. Arterial lactate decreased to 4.8 mmol/L, and central venous saturation was 61%. Left ventricular function improved to an ejection fraction of 53% at day 3. A low dose of dobutamine (2 mcg/kg/min) was continued, and ultrafiltration rate was progressively increased to 250 mL/h.

**Figure 2:**
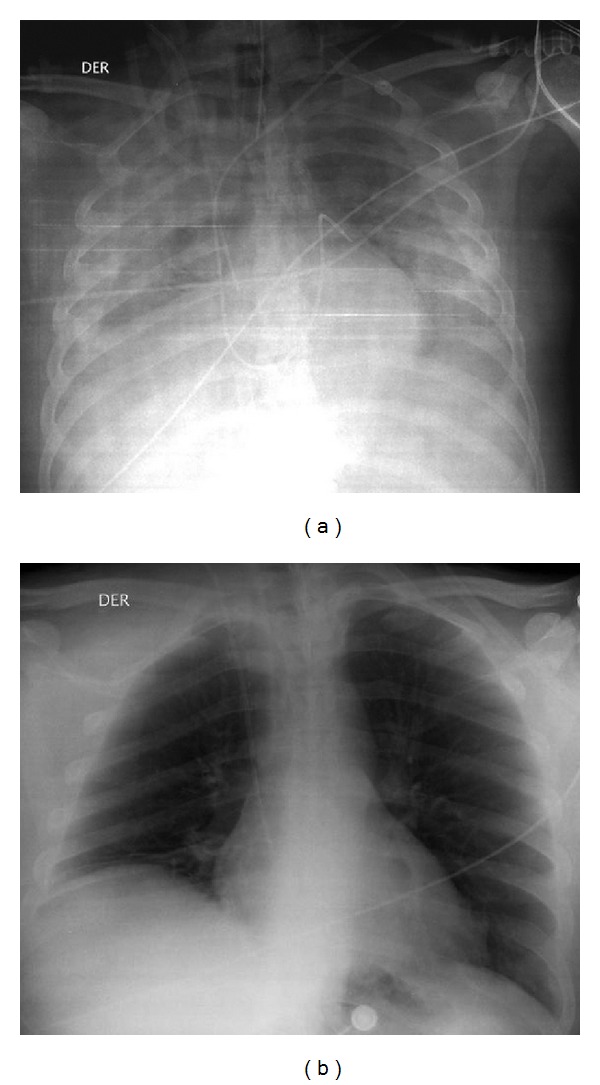
Chest imaging evolution. Chest X-ray on day 2 shows opaque lung fields (a) with resolution of infiltrates an normalization of aeration a week later (b).

In the fifth day since admission, ECMO was removed, and the patient continued on mechanical ventilation, achieving a PaO_2_/FiO_2_ ratio of 180 and OI of 10,5 with PEEP 10 cm of water and FiO_2_ of 60%. Four days later he was extubated and supported by noninvasive ventilation. Chest X-ray revealed a significant reduction of pulmonary infiltrates (Figure 2). During the following days, the patient evidenced the recovery of his respiratory, cardiovascular, and kidney functions and the normalization of the coagulation disorder. He was transferred to intermediate care and then to the general ward. He was discharged after a month of hospitalization requiring physical rehabilitation with no other dysfunctions and normal cognitive functions, resuming his graduate studies.

## 3. Discussion

In this case of Hantavirus-induced cardiopulmonary syndrome, catastrophic respiratory failure and severe shock were treated with prone positioning and vasoactive drugs adjustment to stabilize and transfer the patient as a bridge to ECMO therapy. The treatment in the University Hospital using ECMO was required because both respiratory and cardiac failures were considered potentially reversible, and the patient had not responded to conventional therapy. The transfer of this type of patients is associated with a high incidence of potentially life-threatening complications. In this case, the participation of a trained and experienced team of critical care physicians to rescue the patient was determinant in the success of procedure, after stabilizing the patient, and risk-benefit assessment of employing prone position ventilation as a bridge to definitive therapy.

Transport of critically ill patients has increased in the last years; it was particularly important during the 2009 flu pandemic, when the rapid onset of refractory hypoxemia, together with multisystem organ failure, caused that clinical outcomes largely depended on hospitals capacities and clinicians' expertise to apply sophisticated mechanical ventilatory support and adjunct therapies unavailable in all hospitals. When transferring critical patients is decided, the benefits of more specialized care—the usual reason to transfer—must be weighed against risks of morbidity and mortality related to the procedure [1]. The most common complications of transport are hypotension and hypoxemia, frequently seen in the most severely ill patients receiving higher levels of PEEP or FiO_2_ [2]. Preparations may render the procedure safer if performed by a dedicated team [3].

Clinicians should notice that the rescue therapies used in this case, prone positioning and ECMO, have the potential to cause harm if not implemented in a coordinated manner by an experienced ICU team. Many hospitals may not have adequate numbers of physicians with this expertise or staffing structures to facilitate timely treatment of complications or technical problem resolution at any time. Attempting a high-risk maneuver to accomplish interhospital transportation of critically ill patients should be ruled out if the team does not have sufficient experience, training, or familiarity implementing prone positioning or ECMO. Both strategies have been used in interhospital transportation without major adverse events [4, 5].

Some countries have addressed this issue with national guidelines, coordinating tertiary ICUs to facilitate adequate access to more intensivist-staffed ICUs and implementing dedicated teams and devices as mobile intensive care units “MICUs” that have shown to improve outcomes making transfers a safe procedure [6].

Transporting mechanically ventilated patients in prone positioning due to life-threatening hypoxemia can be achieved successfully with a trained team [5]. In our experience, patients have been transported in prone between different hospital units, from ICU to the department of radiology not only for computed tomography assessment and studies [7] but also for open lung biopsies in the operating room, without adverse events [8]. Noteworthy, before transporting mechanically ventilated patients in prone positioning outside the ICU, the patients have always been assessed by a trained ICU team, carefully reviewed following a detailed protocol, and the maneuvers performed only by experienced intensive care specialists using previously known equipment, such as ventilator and infusion pumps.

Prone ventilation is a feasible and relatively safe therapy that improves oxygenation in ARDS, reduces the risk of ventilator induced lung injury [9], and improves the survival in patients with severe form of ARDS as suggested previously and recently demonstrated in the large clinical trial PROSEVA [10, 11]. Our team has developed experience in prolonged prone ventilation as a protocolized routine therapy for severe ARDS [12], recently applied during the 2009 flu pandemic [13]. In addition, several procedures are performed in prone positioning in ARDS patients in our unit, as the installation of intravascular devices using ultrasound-guided central catheter insertions in the case of central venous and artery pulmonary catheters, fiber optic bronchoscopy, and transesophageal echocardiography; the latter has been reported in other centers [14, 15].

To the best of our knowledge there are no reports transferring patients with Hantavirus cardiopulmonary syndrome in prone position ventilation as a bridge to ECMO. However, there is an interesting case series transporting critically ill patients with severe respiratory and cardiac failures, performed in prone position ventilation, with no complications [5]. These suggest that prone position ventilation is not a contraindication for transport if performed appropriately.

In case reported here, the severe cardiopulmonary syndrome was caused by Hantavirus. Hantavirus is a rodent borne virus from the Bunyaviridae family; its reservoir is the long-tailed pygmy rice rat (*Oligoryzomys longicaudatus*), endemic to some regions of USA, Argentina, Brazil, Chile, Paraguay, Bolivia, Panama, and Canada. Hantavirus infection can lead to different manifestations. Hantavirus cardiopulmonary syndrome is the most severe form, characterized by fever and progressive symptoms. This condition rapidly evolves to cardiogenic shock and pulmonary edema with severe capillary leak. If adequate support is applied, recovery of the most severe dysfunctions occurs in a few days [16]. Thus, Hantavirus infection is a model of hyperacute multisystem failures that can require ECMO as rescue therapy with potential full recovery. Therefore, transfer to centers in which ECMO is available may constitute the difference between and almost certain death and survival. In this context, prone position ventilation could be a lifesaving tool if employed as a bridge to ECMO if other treatments are insufficient to provide safe limits of oxygenation or is not feasible to implement a transfer on ECMO. The key point is adequate monitorization and management on a case-by-case basis by a specialized retrieval team with expertise on rescue therapies [17].

In conclusion, we found that an unstable patient with Hantavirus cardiopulmonary could be transferred without major complications allowing survival. We acknowledge that this is only a single case, but several series show that the participation of a specialized retrieval team, proper patient stabilization before transport, an ambulance with appropriate intensive care equipment, and interhospital transfers of critically ill patients appear to be feasible. Catastrophic cardiorespiratory failures should not be considered contraindications to attempt transfer to tertiary centers if deemed necessary, and prone position ventilation may be the required bridge to do so.
